# Characterization of MgtC, a Virulence Factor of *Salmonella enterica* Serovar Typhi

**DOI:** 10.1371/journal.pone.0005551

**Published:** 2009-05-14

**Authors:** Patricio Retamal, Mario Castillo-Ruiz, Guido C. Mora

**Affiliations:** 1 Departamento de Ciencias Biológicas, Universidad Andrés Bello, Santiago, Chile; 2 Programa de Doctorado Genética Molecular y Microbiología, Pontificia Universidad Católica de Chile, Santiago, Chile; University of Minnesota, United States of America

## Abstract

The MgtC is a virulence factor in *Salmonella* Typhimurium that is required for growth at low-Mg^2+^ concentrations and intramacrophage survival. This gene is codified in a conserved region of the *Salmonella* pathogenicity island 3 (SPI-3), and is also present in the chromosome of other *Salmonella* serovars. In this study we characterized the MgtC factor in *S.* Typhi, a human specific pathogen, by using *mgtC* and SPI-3 mutant strains. We found that MgtC is the most important factor codified in the SPI-3 of *S.* Typhi for growth in low-Mg^2+^ media and survival within human cells. In addition, by using reporter genes we determined that the low-Mg^2+^ concentration, acidic media and PhoP regulator induce *mgtC* expression in *S*. Typhi. We suggest that MgtC is the most important virulence factor codified in the SPI-3 of *S.* Typhi.

## Introduction

The *Salmonella enterica* genome has at least five DNA regions associated with pathogenicity, referred to as the *Salmonella* pathogenicity islands (SPI). One such island, SPI-3, is located in the *selC* locus of *S.* Typhimurium and contains ten ORFs [1], among which some have been experimentally associated with virulence functions of this bacterium. This is the case for the *mgtCB* operon, for which there is evidence of involvement in intramacrophage survival and virulence in mice [2], [3]. This operon is codified in all *Salmonella* serovars in a very conserved SPI-3 region [4]. The *mgtC* sequence seems to encode a virulence factor that has been repeatedly acquired by horizontal gene transfer throughout bacterial evolution, since it has also been associated with virulence in *Mycobacterium tuberculosis* and *Brucella suis* [5]–[7]. MgtC is a protein of unknown function of about 25 kDa in size. In *S. enterica* serovar Typhimurium (*S.* Typhimurium), the experimental evidence suggests that MgtC participates in adaptation to low-Mg^2+^ environments, supporting bacterial invasion and proliferation in macrophages [2]. Although is co-transcribed with *mgtB*, which encodes a Mg^2+^ transporter, MgtC is not required for MgtB function [8]. Indeed, a recently described polypeptide encoded by the *mgtCB* operon, named MgtR, promotes MgtC degradation by a bacterial protease, acting as a negative feedback that limits the amount of MgtC under certain conditions [9]. In addition, it has been shown that the two-component system PhoP-PhoQ induces the expression of *mgtC*, in response to low Mg^2+^ levels and acidic pH [8], [10].

Another SPI-3 gene involved in bacterial pathogenicity is *misL*, which encodes an autotransporter protein involved in the adhesion of *S.* Typhimurium to the extracellular matrix in mice and chicks, thereby acting as an intestinal colonization factor [11], [12]. It has also been shown that *marT*, another sequence present in SPI-3, encodes a transcriptional regulator that induces the expression of *misL* [13]. There is no additional information on other SPI-3 ORFs, all of them remaining until now as sequences encoding conserved hypothetical proteins with unknown function [14].

*S.* Typhimurium is a wild host range serovar and has been extensively studied in a murine model of systemic infection to indirectly elucidate some microbiological and immunological traits of typhoid fever in humans [15], a life-threatening and systemic infection caused by the *S. enterica* serovar Typhi (*S.* Typhi). The latter, a human-restricted pathogen, is a facultative intracellular bacterium responsible for significant morbidity and mortality worldwide, and there are an estimated 21.5 million cases per year, most of which occur in developing countries [16].

The aim of this work was to characterize the role of the MgtC factor in the virulence of *S.* Typhi by comparing the growth and survival of *mgtC* and SPI-3 mutant strains in different stressful conditions, and determining the signals and transcriptional regulators that command MgtC expression. We demonstrated that MgtC is the most important factor in *S*. Typhi SPI-3 for bacterial growth in a low-Mg^2+^ environment and for bacterial survival inside human cells. In addition, the PhoP regulator participates in inducing the expression of *mgtC* in *S.* Typhi.

## Materials and Methods

### Bacterial strains and growth conditions

All *Salmonella* Typhi strains used in this study are derived from STH2370, a Chilean clinical isolate described previously [15]. Unless otherwise stated bacteria were grown at 37°C in Luria Bertani (LB) broth or in M9 minimal medium supplemented with either 10 µM or 10 mM MgCl_2_, 0.2% glucose, tryptophan and cysteine (50 µg/mL each). When necessary, the pH was adjusted to pH 7.0 (NaHPO_4_/NaH_2_PO_4_ 25 mM) or 5.0 (citric acid/sodium citrate 0.1 M) and the following antibiotics were added: chloramphenicol (Cam; 20 µg/mL), kanamycin (Kan; 50 µg/mL), ampicillin (Amp; 100 µg/mL) and gentamicin (Gem; 50 µg/mL).

### PCR amplifications and construction of mutant strains

PCR amplifications were performed in a standard volume of 25 µL. Reaction mixes contained 1× PCR buffer, 1.5 mM MgCl_2_, each deoxynucleoside triphosphate (200 µM), primers (1 µM), 100 ng of template DNA, and 2 U of Taq (Fermentas) DNA polymerase. Standard conditions for amplification were an initial step of 95°C for 5 min, 30 cycles of incubation at 96°C for 40 s, 60°C for 40 s, and 72°C for 2 min, followed by a final extension step at 72°C for 10 min. Template *S.* Typhi chromosomal DNA was prepared by the phenol chloroform extraction method [17].

The Δ*mgtC*::FRT (Δ*mgtC*) mutant strain was constructed using the lambda Red recombinase system [18]. Briefly, the Cam^R^ cassette (chloramphenicol resistance, codified in the pKD3 plasmid) was amplified using the primers MGW1 (5′-ATGGAGGAACGTATGTTAATGTTTCCTTATATTTTAAATTTGTAGGCTGGAGCTGCTTCG-3′) and MGW2 (5′-TGACCCACGAGCTCGGCACGAATTTCTTTATAGCCCTGTTCATATGAATATCCTCCTTA-3′). Once the Δ*mgtC::cat* mutant strain was obtained, the Cam^R^ determinant was removed and substituted by the “FRT scar” [18], and the resulting colonies were tested by PCR to confirm the *mgtC* deletion.

The ΔSPI-3::FRT mutant (ΔSPI-3) was constructed with the same procedure, amplifying the Kan^R^ cassette (kanamycin resistance, codified in the pKD4 plasmid) using the primers SPW1 (5′-AACGCAGGCGCTACGTTTGTCGATGCCGTAACTTTCTGAATGTAGGCTGGAGCTGCTTCG-3′) and SPW2 (5′-GCTAAATATAGCACGTACTTATTCTTCCAGAAAAAATGGACATATGAATATCCTCCTTA-3′). Once the ΔSPI-3*::aph* mutant strain was obtained, the Kan^R^ determinant was substituted by the “FRT scar” as described previously [18].

With the mutant strain EG14598 (*S.* Typhimurium 14028s Δ*phoP::cat*) [19], a P22 HT105/1 *int201* phage lysate was made [20] and used for generalized transduction over *S.* Typhi strains.

### Phenotypic analysis of the *S.* Typhi mutant strains

Growth in a Mg^2+^-limiting environment was evaluated as described previously [21] with some modifications. Briefly, an overnight culture grown in M9 minimal media with 10 mM MgCl_2_ was washed three times with Mg^2+^-free medium, diluted 1/200 in culture media containing either 10 µM or 10 mM MgCl_2_, and incubated with shaking at 37°C for different lengths of time. Growth was measured with a spectrophotometer at an optical density of 600 nm (OD_600_).

To evaluate the effect of pH, overnight cultures were grown in LB broth at pH 7, then washed three times with LB at the desired pH (5 or 7), diluted 1/200 in the same medium and incubated with shaking at 37°C for different lengths of time. Growth was measured with a spectrophotometer at OD_600_.

The infection assays using monocytic (U937) and epithelial (HEp-2) human cells were carried out as described previously [15], [22], with the following modifications. Cells were grown in Dulbecco's modified Eagle's medium supplemented with 10% (vol/vol) fetal bovine serum, seeded into 24-well tissue culture plates at a concentration of 10^5^ cells per well, and then incubated at 37°C in 5% CO_2_ until confluent growth was achieved. Later the cells were centrifuged and washed three times with PBS. Approximately 2×10^6^ to 5×10^6^ CFU of exponential-phase (OD_600_, 0.15 to 0.20) anaerobically grown bacteria was pelleted, washed twice with PBS, and resuspended in 1 mL of PBS. Aliquots (100 µL) of bacteria were added to cells at a multiplicity of infection of 50∶1(U937) and 100∶1 (HEp-2). After 1 h of infection, cells were centrifuged and washed three times with PBS, and the medium was replaced with Dulbecco's modified Eagle's medium supplemented with 10% (vol/vol) fetal bovine serum containing gentamicin (200 µg/mL). After additional incubation for 1 and 23 h (times 2 and 24 h respectively) U937 and HEp-2 cells were washed three times with PBS and lysed with 0.5% deoxycholate, and the titers of intracellular bacteria were determined by serial dilution of cell lysates on agar plates. The percentage of survival was calculated at 2 h considering the initial inoculate as 100%, and at 24 considering the CFU counted at 2 h as 100%.

### β-Galactosidase assay

The *mgtC* promoter activity was evaluated by a transcriptional fusion to the Lac reporter, as described previously [23], [24] using the pCE36 plasmid. β-Galactosidase activity was measured by a modification of the Miller's method [25]. Fifty microliters of the bacterial culture were suspended in 950 µL of Z buffer (60 mM Na_2_HPO_4_, 40 mM NaH_2_PO_4_, 10 mM KCl, 1 mM MgSO_4_, 50 mM β-mercaptoethanol, pH 7.0). Bacteria were permeabilized with 10 µL chloroform and 10 µL 0.1% SDS, incubated at 30°C for 10 min, and 200 µL of o-nitrophenyl-β-D-galactopyranoside (4 mg/mL) was added. Reactions were stopped by addition of 500 µL 1 M Na_2_CO_3_. β-Galactosidase activity was calculated in Miller units, using the formula 10^3^×(OD_420_−1.75×OD_550_)/(mL×min×OD_600_).

### RNA isolation and RT-PCR

Total RNA was extracted and purified using Trizol and was treated with RNase-free DNase I (amplification grade; Gibco-BRL). RT-PCR was performed with 500 ng of DNase-treated RNA using the Superscript reverse transcriptase (Invitrogen). Amplification was performed for 30 cycles (94°C for 40 s, 55°C for 40 s, and 72°C for 1.5 min, followed by a 10 min extension at 72°C). The primers used were RTMGC1 (5′-TCGGCGTGTTATGCGGCTTA-3′), RTMGC2 (5′-AGCCCTGTTCCTGAGCGGGG-3′) and RTMGB2 (5′-CACGGCGTAACGGGAGCCAG-3′) corresponding to an internal region of the *mgtC* (RTMGC1 and RTMGC2) and *mgtC*-*mgtB* (RTMGC1-RTMGB2) sequences. In addition, the universal primers 8F and 1498R were used to amplify 16S rRNA [15]. Genomic DNA served as a positive control, and DNase-treated RNA that had not been reverse transcribed was used as a negative control. The PCR product was electrophoresed on 1% agarose gels and stained with ethidium bromide.

### MgtC epitope tagging and immunoblot assay

A translational fusion of three copies of FLAG epitope (3×FLAG) with the MgtC sequence was constructed using the method described by Uzzau et al. [26]. The 3×FLAG epitope codified on the pSUB11 plasmid was amplified using the primers FMG1 (5′-CGATAATATCACCGCAATTCACTGGAGCATTGATAGTCAAGACTACAAAGACCATGACGG-3′) and FMG2 (5′-ACTGACCCCTGCCAGTGCCATCAGAACGTAAATAAACGGGCATATGAATATCCTCCTTAG-3′). Once inserted immediately preceding the translation stop signal, the *mgtC*-3×*flag* fusion was confirmed by PCR and the functionality of the protein was verified by the growth of the strain in low-Mg^2+^ medium.

The MgtC epitope tagging was detected by an immunoblot assay using the anti-FLAG M2 monoclonal antibody (Sigma), as previously described [24]. After the samples were resuspended in 1 mL of 100 mM Tris–HCl (pH 8) and sonicated, the total protein was quantified by the Bradford method. The SDS-PAGE was made using 10 ng of total protein per sample.

### Statistical analysis

Statistical analysis was performed using the one way ANOVA and Student's t-test for independent samples. Values of P<0.05 were considered significant. These tests were performed using Microsoft Excel® software.

## Results

### 1. MgtC is required for *S.* Typhi growth in a low-Mg^+2^ medium

**Figure 1:**
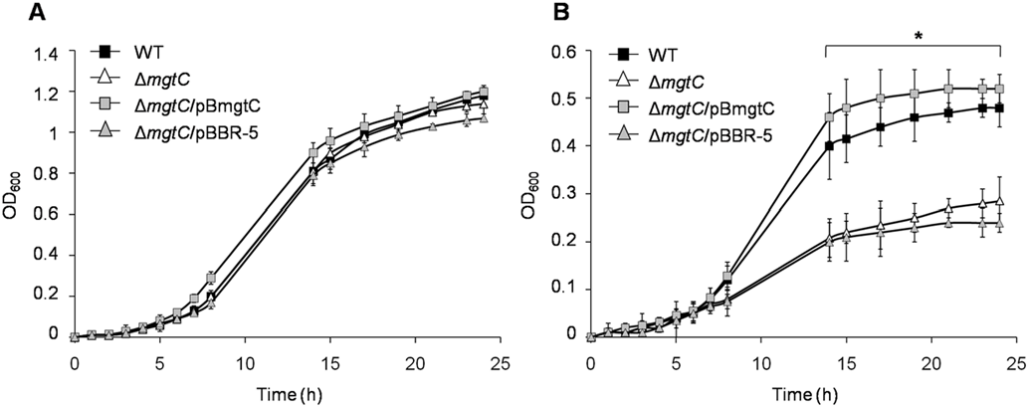
MgtC is necessary for growth at a low-Mg^2+^ concentration. Strains WT (STH2370 wild type), Δ*mgtC* (*mgtC^−^*), Δ*mgtC*/pBmgtC (*mgtC* ^+^) and Δ*mgtC*/pBBR-5 (*mgtC^−^*) were grown in M9 minimal medium supplemented with 10 mM (A) or 10 µM (B) MgCl_2_. The OD_600_ was measured at the indicated times. Values represent the mean of three independent experiments ±SD (*p<0.05).

To elucidate the role of *mgtC* in *S.* Typhi, we investigated growth in low-Mg^+2^ minimal media as evaluated previously in *S.* Typhimurium [2], [8]. The Mg^2+^ concentrations used were 10 µM or 10 mM, representing the intracellular and extracellular environment respectively [2], [27]. At the 10 µM concentration, the Δ*mgtC* mutant strain grew significantly less than the wild type strain (p<0.05), reestablishing its phenotype when complemented with *mgtC* cloned in pBBR-5 plasmid. In contrast, at 10 mM Mg^2+^ there was no difference among the tested strains (Fig. 1A and 1B). These results are in accordance with reports of the role of MgtC in the virulence of *S.* Typhimurium [2], [8] and other bacteria [5], [6].

### 2. MgtC is required for growth of *S.* Typhi within epithelial and monocytic human cells

**Figure 2:**
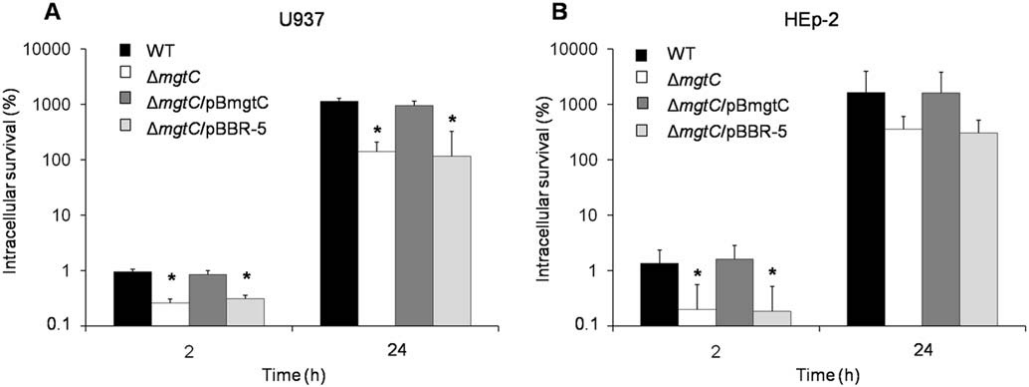
MgtC has an important role in the growth of S. Typhi within human cells. Infection assays in U937 (A) and HEp-2 (B) human cells using WT (STH2370 wild type), Δ*mgtC* (*mgtC^−^*), Δ*mgtC*/pBmgtC (*mgtC* ^+^) and Δ*mgtC*/pBBR-5 (*mgtC^−^*) strains. Culture cells were infected at a MOI of 50∶1 (U937) and 100∶1 (HEp-2), respectively. Colonies were counted at time 2 h and 24 h and expressed as a percentage of intracellular survival. Values represent the mean of at least three independent experiments ±SD (*p<005).

To verify the role of MgtC in the intracellular survival of *S.* Typhi, we tested the Δ*mgtC* strain in infection assays using both HEp-2 epithelial and U937 monocytic cell lines. Two post-infection times were evaluated, 2 h and 24 h, representing the early and late survival abilities respectively. As expected, MgtC is required for infection of monocytic cells, with significant differences (p<0.05) among wild type and Δ*mgtC* strains (Fig. 2A). Remarkably, inside HEp-2 epithelial cells there was a significant impairment (p<0.05) in the invasive phenotype of the Δ*mgtC* mutant strain (Fig. 2B), suggesting that MgtC participates during the infection of this kind of human cell.

### 3. MgtC reestablishes the wild type phenotype of a SPI-3 mutant strain both in low Mg^2+^ media and inside monocytic cells

**Figure 3:**
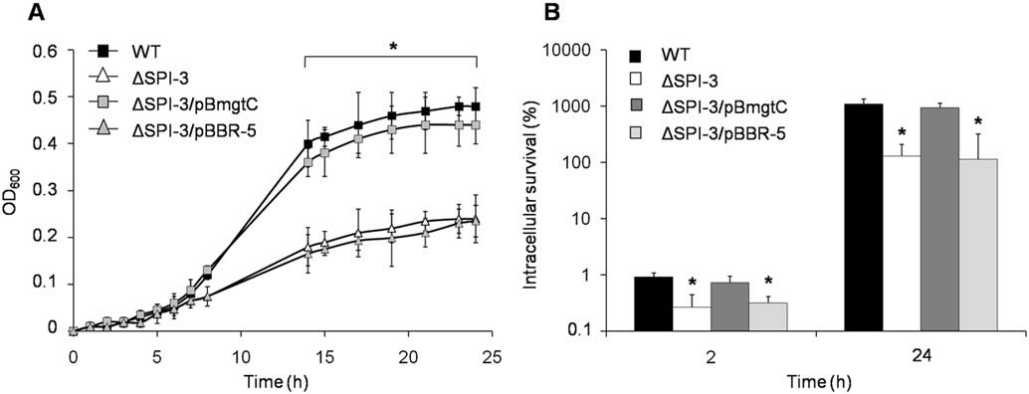
MgtC can restore the WT phenotype in a SPI-3 mutant strain. Strains WT (STH2370 wild type), ΔSPI-3 (SPI-3*^−^*), ΔSPI-3/pBmgtC (*mgtC* ^+^) and ΔSPI-3/pBBR-5 (SPI-3*^−^*). (A) For growth in 10 µM MgCl_2_ strains were incubated in M9 minimal medium and the OD_600_ was measured at the indicated times. (B) U937 cells were infected at a MOI of 50∶1. Colonies were counted at time 2 h and 24 h and expressed as a percentage of intracellular survival. Values represent the mean of at least three independent experiments ±SD (*p<0.05).

Previously it has been shown that MgtC can restore the wild type intramacrophage survival phenotype of a *S*. Typhimurium *mgtCB* mutant strain [2]. In *S.* Typhi we wanted to determine whether MgtC is required for intracellular survival and growth at low-Mg^2+^ concentrations in the context of a SPI-3 deletion. Therefore, we constructed the ΔSPI-3 mutant strain, the complemented ΔSPI-3/pBmgtC (*mgtC* ^+^) and ΔSPI-3/pBBR5 (*mgtC^−^*) strains, and repeated the assays of growth in low Mg^2+^ media (Fig. 3A) and survival inside human monocytic cells (Fig. 3B). The results suggest that MgtC can restore the phenotypes observed in a SPI-3 mutant strain by itself and can be considered the most important product codified on the *S.* Typhi SPI-3 island for bacterial response to those experimental conditions.

### 4. The PhoP regulator controls *mgtC* expression in a low Mg^2+^ and acidic environment

**Figure 4:**
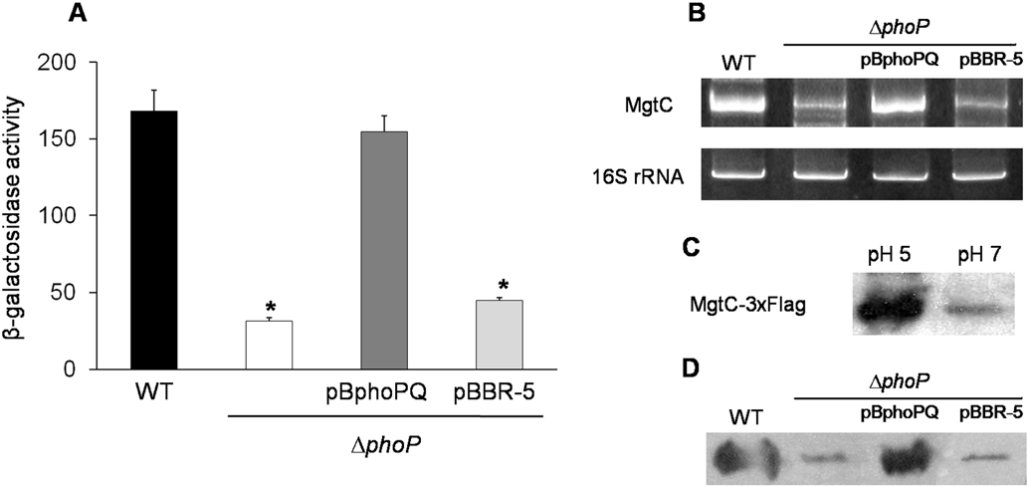
The PhoP regulator controls the expression of *mgtC* in *S*. Typhi. The strains used were WT (STH2370 wild type), Δ*phoP* (*phoP*::*cam*), Δ*phoP*/pBphoPQ (*phoPQ* ^+^) and Δ*phoP*/pBBR-5 (*phoPQ* ^−^). The cultures were grown in acidic LB medium (pH 5) for 5 h, and the samples taken had an OD_600_ ranging from 0.3 to 0.6. The expression of *mgtC* was evaluated using three methods: A, β-Galactosidase assay with strains carrying the reporter *lacZ* gene downstream of the *mgtC* promoter. Values represent the mean of at least three independent experiments ±SD (*p<0.05). B, RT-PCR assay. C, Immunoblot method using a STH2370 MgtC-3×Flag epitope-tagged strain grown on LB broth at both pH 5 and pH 7. D, Immunoblot method using strains carrying the MgtC-3×Flag epitope tag and grown in acidic LB medium (pH 5) for 5 h.

In *S.* Typhimurium, the PhoP-PhoQ two-component system regulates the expression of many genes when bacteria are exposed to the intracellular environment, including SPI-2 and SPI-3 associated effectors [10], [19]. The signals sensed by this regulatory system are the extracellular pH and Mg^2+^ concentrations. PhoQ is the sensor component that phosphorylates the PhoP regulator, which then modifies gene expression. By using a STH2370 Δ*phoP::cat* mutant strain (Δ*phoP*), we examined whether PhoP regulates MgtC in *S.* Typhi. The results obtained by β-galactosidase and RT-PCR assays show that *mgtC* transcription is induced in a *phoP*-dependent manner by either low Mg^2+^ (10 µM, data not shown) or pH 5 (Fig. 4A and 4B). Moreover, immunoblot assay shows an increase in the MgtC levels under the same conditions (Fig. 4C and 4D), a finding that differs from the reported situation in *S*. Typhimurium where MgtC translation is not detected in many hours [8], [9].

## Discussion

In previous reports the *Salmonella* SPI-3 island has been associated with intramacrophage invasion by supporting survival when Mg^2+^ is scarce, a condition that seems a common strategy of the host to avoid the growth of intracellular bacteria [1], [2], [8]. Mg^2+^ is a divalent ion essential for living organisms that works as a regulator and co-factor in many proteins, stabilizing membranes, ribosomes and other cellular structures [28]. *Salmonella* contains several transport systems, both inducible and constitutive, that have functional complementarities with the aim of adjusting the Mg^2+^ concentration in different environmental conditions [29], [30]. In addition, these systems are controlled by transcriptional and post-transcriptional regulatory networks to maintain strict control of the Mg^2+^ balance [31], [32], stabilizing its concentrations as required for biological processes in *Salmonella*. In this context, MgtC seems to be the most important SPI-3 factor that supports the survival and growth of *Salmonella* in low-Mg^2+^ concentrations, as observed in previous reports for *S.* Typhimurium and in this work with *S.* Typhi. This factor is codified in a SPI-3 conserved region [4] and probably exerts the same, although yet unknown, function in all *Salmonella* serovars.

In this work, the decreased ability to survive within human monocytic cells observed with a *S.* Typhi ΔSPI-3 strain could be overcome with an *mgtC*-containing plasmid, which restored the wild type phenotype at 2 and 24 hours post-infection. This means that MgtC is a virulence factor playing a major role that is not supplied by any other bacterial factor codified either inside SPI-3 or in the entire chromosome of *Salmonella*.

A characteristic phenotype associated with S. Typhi MgtC was demonstrated by the early survival phenotype inside HEp-2 epithelial cells, in which the *S.* Typhi *mgtC* mutant strain showed a significant lower survival (p<0.05) than the wild type strain (Fig. 2B). This difference has not been reported previously in any *Salmonella* serovar and suggests that *S.* Typhi requires the MgtC function from the initial infective phase, when it colonizes the intestinal epithelium. Whether this requirement responds to particular conditions during bacterial entry or when bacteria are inside human epithelial cells are questions that remain to be elucidated.

However, these findings are indicating that both epithelial and monocytic human cells represent a variety of conditions that require the MgtC function for the bacterial survival. This hypothetical “multi-requirement” of MgtC is in accordance to several reports suggesting a connection of this virulence factor with the structural stability of Mg^2+^ channels in the bacterial cell membrane [7], linked to survival in Mn^+2^ depleted environments, or modifying the membrane potential of the host cell and affecting the host-pathogen interaction [33], [34]. Since single amino acid substitutions can affect one role of MgtC without affecting others [35], it seems possible to assume a diversity of functions in which this internal membrane protein participates during infection. In addition, MgtC is important in other bacterial species that are able to invade, live and proliferate within host cells, as for *Mycobacterium tuberculosis*, *Brucella melitensis* and *Yersinia pestis* [5], [7].

The expression assays showed that *mgtC* transcription and translation are induced at low Mg^2+^ concentrations and acidic pH, and that PhoP is the global regulator that participates in this process. These signals stimulate the expression of many genes associated with pathogenicity by inducing the PhoP-PhoQ system [10], and MgtC of *S.* Typhi is one of these. In addition, *mgtC* and *mgtB* are co-transcribed in *S.* Typhi (data not shown), suggesting the almost identical functionality of these sequences between *S.* Typhimurium and *S.* Typhi, and probably in all *Salmonella* serovars that contain the *mgtCB* operon in their chromosomes. Previously it has been shown in *S*. Typhimurium that MgtR induces the degradation of MgtC but not of MgtB, resulting in a downregulation of MgtC when the operon is expressed [9]. Our results suggest that in *S*. Typhi this regulation could be different, since MgtC is detected after acidic or low magnesium stimuli.

In conclusion, in this work we determined that MgtC in *S.* Typhi represents a mechanism of pathogenicity codified inside the SPI-3 that has a relevant role in the intracellular survival of bacteria, induced by the PhoP global regulator in response to a low-Mg^2+^ concentration and acidic pH. These findings suggest that MgtC is a key factor in most, if not all, pathogenic *Salmonella* serovars.

## References

[1] Blanc-Potard AB, Solomon F, Kayser J, Groisman EA (1999). The SPI-3 pathogenicity island of *Salmonella enterica*.. J Bacteriol.

[2] Blanc-Potard AB, Groisman EA (1997). The *Salmonella selC* locus contains a pathogenicity island mediating intramacrophage survival.. Embo J.

[3] Smith RL, Kaczmarek MT, Kucharski LM, Maguire ME (1998). Magnesium transport in *Salmonella* typhimurium: regulation of *mgtA* and *mgtCB* during invasion of epithelial and macrophage cells.. Microbiology.

[4] Amavisit P, Lightfoot D, Browning GF, Markham PF (2003). Variation between pathogenic serovars within *Salmonella* pathogenicity islands.. J Bacteriol.

[5] Buchmeier N, Blanc-Potard A, Ehrt S, Piddington D, Riley L (2000). A parallel intraphagosomal survival strategy shared by *Mycobacterium tuberculosis* and *Salmonella enterica*.. Mol Microbiol.

[6] Lavigne JP, O'Callaghan D, Blanc-Potard AB (2005). Requirement of MgtC for *Brucella suis* intramacrophage growth: a potential mechanism shared by *Salmonella enterica* and *Mycobacterium tuberculosis* for adaptation to a low-Mg^2+^ environment.. Infect Immun.

[7] Blanc-Potard AB, Lafay B (2003). MgtC as a horizontally-acquired virulence factor of intracellular bacterial pathogens: evidence from molecular phylogeny and comparative genomics.. J Mol Evol.

[8] Moncrief MB, Maguire ME (1998). Magnesium and the role of MgtC in growth of *Salmonella* typhimurium.. Infect Immun.

[9] Alix E, Blanc-Potard AB (2008). Peptide-assisted degradation of the *Salmonella* MgtC virulence factor.. Embo J.

[10] Groisman EA (2001). The pleiotropic two-component regulatory system PhoP-PhoQ.. J Bacteriol.

[11] Dorsey CW, Laarakker MC, Humphries AD, Weening EH, Baumler AJ (2005). *Salmonella enterica* serotype Typhimurium MisL is an intestinal colonization factor that binds fibronectin.. Mol Microbiol.

[12] Morgan E, Campbell JD, Rowe SC, Bispham J, Stevens MP (2004). Identification of host-specific colonization factors of *Salmonella enterica* serovar Typhimurium.. Mol Microbiol.

[13] Tukel C, Akcelik M, de Jong MF, Simsek O, Tsolis RM (2007). MarT activates expression of the MisL autotransporter protein of *Salmonella enterica* serotype Typhimurium.. J Bacteriol.

[14] Chaudhuri RR, Khan AM, Pallen MJ (2004). coliBASE: an online database for *Escherichia coli*, *Shigella* and *Salmonella* comparative genomics.. Nucleic Acids Res.

[15] Bucarey SA, Villagra NA, Martinic MP, Trombert AN, Santiviago CA (2005). The *Salmonella enterica* serovar Typhi *tsx* gene, encoding a nucleoside-specific porin, is essential for prototrophic growth in the absence of nucleosides.. Infect Immun.

[16] Pang T, Levine MM, Ivanoff B, Wain J, Finlay BB (1998). Typhoid fever–important issues still remain.. Trends Microbiol.

[17] Sambrook J, Fritsch F, Maniatis T (1989). Molecular Cloning: a Laboratory Manual.

[18] Datsenko KA, Wanner BL (2000). One-step inactivation of chromosomal genes in *Escherichia coli* K-12 using PCR products.. Proc Natl Acad Sci U S A.

[19] Bijlsma JJ, Groisman EA (2005). The PhoP/PhoQ system controls the intramacrophage type three secretion system of *Salmonella enterica*.. Mol Microbiol.

[20] Schmieger H (1972). Phage P22-mutants with increased or decreased transduction abilities.. Mol Gen Genet.

[21] Tu X, Latifi T, Bougdour A, Gottesman S, Groisman EA (2006). The PhoP/PhoQ two-component system stabilizes the alternative sigma factor RpoS in *Salmonella enterica*.. Proc Natl Acad Sci U S A.

[22] Contreras I, Toro C, Troncoso G, Mora G (1997). *Salmonella* Typhi mutants defective in anaerobic respiration are impaired in their ability to replicate within epithelial cells.. Microbiology.

[23] Ellermeier CD, Janakiraman A, Slauch JM (2002). Construction of targeted single copy lac fusions using lambda Red and FLP-mediated site-specific recombination in bacteria.. Gene.

[24] Bucarey SA, Villagra NA, Fuentes JA, Mora GC (2006). The cotranscribed *Salmonella enterica* sv. Typhi *tsx* and *impX* genes encode opposing nucleoside-specific import and export proteins.. Genetics.

[25] Miller FD, Hershberger CL (1984). A quantitative beta-galactosidase alpha-complementation assay for fusion proteins containing human insulin B-chain peptides.. Gene.

[26] Uzzau S, Figueroa-Bossi N, Rubino S, Bossi L (2001). Epitope tagging of chromosomal genes in *Salmonella*.. Proc Natl Acad Sci U S A.

[27] Eriksson S, Lucchini S, Thompson A, Rhen M, Hinton JC (2003). Unravelling the biology of macrophage infection by gene expression profiling of intracellular *Salmonella enterica*.. Mol Microbiol.

[28] Maguire ME, Cowan JA (2002). Magnesium chemistry and biochemistry.. Biometals.

[29] Chamnongpol S, Groisman EA (2002). Mg^2+^ homeostasis and avoidance of metal toxicity.. Mol Microbiol.

[30] Smith RL, Maguire ME (1998). Microbial magnesium transport: unusual transporters searching for identity.. Mol Microbiol.

[31] Spinelli SV, Pontel LB, Garcia Vescovi E, Soncini FC (2008). Regulation of magnesium homeostasis in *Salmonella*: Mg^(2+)^ targets the *mgtA* transcript for degradation by RNase E.. FEMS Microbiol Lett.

[32] Cromie MJ, Shi Y, Latifi T, Groisman EA (2006). An RNA sensor for intracellular Mg^(2+)^.. Cell.

[33] Alix E, Blanc-Potard AB (2007). MgtC: a key player in intramacrophage survival.. Trends Microbiol.

[34] Gunzel D, Kucharski LM, Kehres DG, Romero MF, Maguire ME (2006). The MgtC virulence factor of *Salmonella enterica* serovar Typhimurium activates Na^(+)^,K^(+)^-ATPase.. J Bacteriol.

[35] Rang C, Alix E, Felix C, Heitz A, Tasse L (2007). Dual role of the MgtC virulence factor in host and non-host environments.. Mol Microbiol.

